# Estimating age-specific COVID-19 fatality risk and time to death by comparing population diagnosis and death patterns: Australian data

**DOI:** 10.1186/s12874-021-01314-w

**Published:** 2021-06-21

**Authors:** Ian C. Marschner

**Affiliations:** Trials Centre, National Health and Medical Research Council Clinical Trials Centre, The University of Sydney, Sydney, NSW 2006 Australia

**Keywords:** Age-specific incidence, Case fatality, COVID-19, Deconvolution, Mortality, Surveillance data

## Abstract

**Background:**

Mortality is a key component of the natural history of COVID-19 infection. Surveillance data on COVID-19 deaths and case diagnoses are widely available in the public domain, but they are not used to model time to death because they typically do not link diagnosis and death at an individual level. This paper demonstrates that by comparing the unlinked patterns of new diagnoses and deaths over age and time, age-specific mortality and time to death may be estimated using a statistical method called deconvolution.

**Methods:**

Age-specific data were analysed on 816 deaths among 6235 cases over age 50 years in Victoria, Australia, from the period January through December 2020. Deconvolution was applied assuming logistic dependence of case fatality risk (CFR) on age and a gamma time to death distribution. Non-parametric deconvolution analyses stratified into separate age groups were used to assess the model assumptions.

**Results:**

It was found that age-specific CFR rose from 2.9% at age 65 years (95% CI:2.2 – 3.5) to 40.0% at age 95 years (CI: 36.6 – 43.6). The estimated mean time between diagnosis and death was 18.1 days (CI: 16.9 – 19.3) and showed no evidence of varying by age (heterogeneity P = 0.97). The estimated 90% percentile of time to death was 33.3 days (CI: 30.4 – 36.3; heterogeneity P = 0.85). The final age-specific model provided a good fit to the observed age-stratified mortality patterns.

**Conclusions:**

Deconvolution was demonstrated to be a powerful analysis method that could be applied to extensive data sources worldwide. Such analyses can inform transmission dynamics models and CFR assessment in emerging outbreaks. Based on these Australian data it is concluded that death from COVID-19 occurs within three weeks of diagnosis on average but takes five weeks in 10% of fatal cases. Fatality risk is negligible in the young but rises above 40% in the elderly, while time to death does not seem to vary by age.

**Supplementary Information:**

The online version contains supplementary material available at 10.1186/s12874-021-01314-w.

## Background

Understanding the natural history of an infectious disease is critical for modelling, intervention and control. A key element of the natural history is mortality, which includes both the risk of death among infected individuals and the time to death among fatal cases. For COVID-19, the risk of death is known to have a steep age gradient [1–3] and various studies in selected cohorts that link onset and death at an individual level have been used to obtain information about the time to death [2–6]. Nonetheless, there has not yet been a comprehensive model of age-specific COVID-19 mortality developed using a data source that captures complete information within a specific population.

In this paper we analyse age-specific surveillance data from Australia on new COVID-19 case diagnoses and deaths over time. Despite their wide availability globally, surveillance data are not used for assessing time to death, because they do not link time of diagnosis and time of death at an individual level. We overcome this complexity by using an analysis method called deconvolution. This is an analysis method that has been used for other purposes during the COVID-19 pandemic [7–9] and has a long history for infectious disease surveillance data dating back to the early analyses of the AIDS pandemic [10–12]. The advantage of using deconvolution to assess mortality is that it does not require prospective follow-up data that links information on diagnosis and death at an individual level. Instead, the method relies on comparing the unlinked patterns of new diagnoses and deaths within a population.

A deconvolution analysis begins by modelling the daily observed death counts in terms of a combination of the daily observed case counts and the unknown fatality distribution, which is a probability distribution specifying both the probability of death and the distribution of the time to death. Deconvolution then involves separating the fatality distribution from the observed counts by choosing an estimate that best aligns the modelled death counts to the observed death counts. Using age-specific data, this yields an age-specific estimate of the COVID-19 fatality distribution, which specifies the probability of death and the distribution of the time to death, both as functions of age.

Our primary analysis involves fitting a mortality model to age-specific COVID-19 surveillance data from the state of Victoria, Australia. This analysis leads to new insights about age-specific COVID-19 mortality and provides useful information that is relevant to mortality assessment in other countries. In particular, assessments of case fatality risk (CFR) often use an estimate of the distribution of time between diagnosis and death to adjust CFR estimates for censoring of the death time in recent cases [5, 8, 13–15]. Furthermore, a model of age-specific mortality provides information that can inform the calibration of mathematical models of COVID-19 transmission dynamics [16, 17]. Our fitted model is a useful aid for these and other epidemiological activities central to monitoring and control of COVID-19.

## Methods

### Surveillance data

The study used surveillance data from the state of Victoria, Australia. The Department of Health and Human Services in Victoria provides a line listing of the date and the 10-year age group of all confirmed cases for public download [18]. This was used to construct the daily age-specific case series stratified by age group. The corresponding line listing for deaths is not provided directly, however, the department makes a daily announcement about the age group of any new COVID-19 deaths occurring in the state, from which it is possible to construct a line listing of the age group and date of all deaths in the state. Such a line listing is available for public download [19], and was used to construct the daily age-specific death series with age group classified into the same 10-year categories as the case data.

According to standard definitions of elimination [20], COVID-19 was eliminated from Victoria when the state experienced 42 consecutive days with zero new cases, from 30 October – 10 December, 2020. It was present again from 11 December when a new outbreak began from an imported case. The Victorian data series ending on 10 December may therefore be viewed as a completed outbreak. Since elimination of the virus from a population is unusual globally, this makes the Victorian data series a valuable resource for studying fatality risk. At any given time, most populations have active cases for which the time of death has yet to be observed and is therefore right-censored. In contrast, the Victorian population as of 10 December had no active cases, which means that the crude case fatality risk, calculated as the ratio of total deaths to total cases, is not subject to the usual underestimation bias arising from right-censoring of death times in active cases [21]. This situation makes estimation of the age-specific fatality distribution using the Victorian data series more reliable than would be the case for an active outbreak. Accordingly, this study used data over the period 25 January 2020, when the first case occurred, through to 10 December 2020.

### Fatality distribution

The primary focus of the analysis is the fatality distribution, which is a probability distribution that captures information about both the probability of death and the time to death among fatal cases. The fatality distribution is technically a sub-distribution, which is a probability distribution with total probability mass less than one, due to the fact that not all COVID-19 infections lead to death. Sub-distributions are common in competing risks analysis, such as cause-specific mortality, where individuals can only experience one of a number of possible event types [22]. In the context of COVID-19, not all infections lead to death because there is a competing endpoint of recovery. This means that the fatality distribution has total probability mass equal to the probability of death, which is less than one.

The age-specific fatality distribution is defined in terms of two outcomes, the time $T$ from diagnosis to death (in days) and the endpoint $E$ of the infection, which is either “Death” or “Recovery”. The fatality distribution is then the cumulative sub-distribution function of $T$ when $E=$ Death, which is also referred to as the cumulative incidence function. In an age-specific context, this function depends on both the time $t$ since diagnosis and the age $a$ at diagnosis:

$$F\left(t,a\right)=\mathrm{Pr}\left(T\le t , E={\text{Death}} \right| {\text{age}}=a).$$

Using standard conditional probability rules, this leads to the basic relationship that will be used to specify a model of the fatality distribution:

1 $$F(t,a)=\mathrm{Pr}\left(T\le t \right| E= \text{Death }, {\text{age}}=a)\times \mathrm{Pr}\left(E={\text{Death}} \right|\text{age }=a)$$

The age-specific fatality distribution specified by Eq. (1) is a fundamental quantity that allows a range of information about COVID-19 mortality to be obtained. The risk of mortality within $t$ days of diagnosis for an individual of age a is simply $F(t,a)$. The age-specific case fatality risk (CFR), also called the case fatality rate or ratio, is specified by

$${\text{CFR}}\left(a\right)=\underset{t\to \infty }{\mathrm{lim}}F\left(t,a\right)$$

The cumulative distribution function of the time to death among cases with $E={\text{Death}}$ is

$${F}_{D}\left(t,a\right)=\frac{F\left(t,a\right)}{{\text{CFR}}\left(a\right)}$$

This is a genuine probability distribution with total probability mass equal to one, because it specifies the distribution of time to death only among fatal cases. Summary measures from this distribution, such as the mean, standard deviation and percentiles, provide statistical summaries of the time to death among fatal cases.

### Model assumptions

The definition of the fatality distribution in Eq. (1) requires modelling assumptions to fit it to data. We will assume a parametric model with a gamma probability distribution for the time to death among fatal cases, and a logistic (sigmoidal) relationship between the case fatality risk and age. A gamma time to death distribution has been the most common choice in past analyses of linked data on COVID-19 mortality [5, 6, 15], although other choices have also been explored, including the log-normal and Weibull distributions [6]. Since all three distributions are right-skewed, unimodal and positive it is unlikely that the data will possess sufficient sensitivity to differentiate between them. Simulation results discussed below and reported in the Supplementary Information (Additional File 1) support this. Nonetheless, alternatives to the gamma distribution could be incorporated into the methods described below, particularly if poor model fit was detected. Likewise, the choice of a logistic relationship between case fatality and age is a natural one, although in principle other sigmoidal relationships could also be explored. As will be shown, the good model fit for the current data set provides a compelling justification for the gamma-logistic model.

The logistic age-specific CFR model is specified in terms of the parameters ${L}_{0}$, ${L}_{1}$ and ${L}_{2}$ as

2 $${\text{CFR}}\left(a\right)=\mathrm{Pr}\left(E={\text{Death}} \right|\text{age }=a)=\frac{{L}_{0}}{1+{\text{exp}}({L}_{1}+{L}_{2}a)}$$

In the logistic model (2) the parameter ${L}_{0}$ specifies the upper limit of the CFR as age increases, while ${L}_{0}$ and ${L}_{1}$ govern the location and speed with which the CFR increases with age. The gamma distribution for the time to death may be specified in terms of the parameters ${G}_{1}$ and ${G}_{2}$ as

3 $${F}_{D}\left(t,a\right)=\frac{{\int }_{0}^{t}{G}_{1}^{{G}_{2}}{u}^{{G}_{2}-1}\mathrm{exp}\left(-{G}_{1}u\right)du}{{\int }_{0}^{\infty }{G}_{1}^{{G}_{2}}{u}^{{G}_{2}-1}\mathrm{exp}\left(-{G}_{1}u\right)du}$$

Using the two components of the fatality distribution model specified in Eqs. (2) and (3), together with Eq. (1), we obtain the parametric model in terms of the parameters $({L}_{0},{L}_{1},{L}_{2},{G}_{1},{G}_{2})$

4 $$F\left(t,a\right)={F}_{D}\left(t,a\right)\times {\text{CFR}}\left(a\right)$$

In the gamma model specified by (3), the parameter ${G}_{1}$ is the rate parameter for which larger values correspond with a smaller mean, while the parameter ${G}_{2}$ is the shape parameter for which larger values correspond with a larger mean. Both ${G}_{1}$ and ${G}_{2}$ may depend on the age $a$ if the time to death depends on age, however, we will also explore models in which the distribution of the time to death is constant over age, so that ${F}_{D}\left(t,a\right)={F}_{D}(t)$, and the model of the fatality distribution is then the product of a time-dependent term and an age-dependent term.

### Deconvolution

Deconvolution is a statistical analysis method that has been used for COVID-19 and other pandemics, for the purpose of reconstructing infection incidence based on the observed case series and an assumed known incubation period distribution [7, 11]. This process is called back-projection or back-calculation. Such analyses use the fact that the infection incidence and the case series are linked by the incubation period distribution, so that if the case series and the incubation distribution are known, then the infection incidence can be reconstructed.

In the current deconvolution analysis, we use an adaptation of these methods applied to the situation where we have a case series and a death series that are linked by an unknown fatality distribution. Since the case series and the death series are observed, we can use them to estimate the unknown fatality distribution. Algorithms for this type of analysis were recently presented for CFR assessment, using adaptations of related methods developed previously for AIDS [23–25]. Software is also available to implement these algorithms, in the R package covidSurv [26].

The deconvolution analysis is based on the convolution relationship linking the death series and the case series through the fatality distribution. To define this relationship we use a discrete time scale$d=\mathrm{1,2},\dots , n$, corresponding to the days on which counts are available, and discrete ages $a={a}_{1},{a}_{2},\dots , {a}_{m}$, corresponding to the midpoints of $m$ age groups. The case and death data are then the $n\times m$ matrices $\{{C}_{di};d=1, \dots , n, i=1, \dots , m\}$ and $\{{D}_{di};d=1, \dots , n, i=1, \dots , m\}$, where ${C}_{di}$ and ${D}_{di}$ are the number of cases and deaths on day $d$ in age group $i$. Then, using a discretised version of the fatality distribution

5 $${P}_{di}=F\left(d,{a}_{i}\right)-F\left(d-1,{a}_{i}\right)=\mathrm{Pr}\left(T=d , E={\text{Death}} \right| \text{age group}=i)$$

the convolution relationship for the expected (mean) number of deaths is

6 $$E\left({D}_{di}\right)=\sum_{u=1}^{d}{C}_{ui}{P}_{d-u+1, i}$$

Since the ${C}_{ui}$ and ${D}_{di}$ values are observed, this is effectively a linear regression model that can be used estimate the unknown ${P}_{di}$ coefficients, which specify the fatality distribution. This process of disaggregating the unknown ${P}_{di}$ coefficients from the observed ${C}_{ui}$ values is called deconvolution. The link between the general convolution relationship (6) and the parametric model specified by Eqs. (2), (3) and (4), is the definition of ${P}_{di}$ in (5). In particular, if the fatality distribution $F(d,a)$ is specified using the parametric model in Eqs. (2), (3) and (4), then the model (6) is a function of the parameters and fitting the model (6) leads to estimates of the parameters. On the other hand, if the probabilities ${P}_{di}$ are left unrestricted without any parametric assumptions, then (6) defines a high dimensional non-parametric model for the fatality distribution. As described below, both of these approaches will be used, with the parametric analysis being the primary analysis.

Although the model has a linear regression structure, the need for non-negativity constraints on the probabilities ${P}_{di}$, together with the large number of coefficients, necessitate specialised numerical algorithms to optimally fit the expected values $E\left({D}_{di}\right)$ to the observed values ${D}_{di}$. For the non-parametric model, the core computational method is the same basic algorithm used in back-projection of infection counts [7–9], which is an iterative procedure developed to fit high-dimensional non-negative linear Poisson regression models [23, 27]. Such algorithms are available within the R package covidSurv [26] which imports the nnpois function from the package addreg to implement the core algorithm [28]. For the parametric model the analysis is a standard maximum likelihood analysis which can be achieved using in-built omnibus optimisation routines available in R. The approach used is to first maximise a profile likelihood in terms of the gamma distribution parameters, followed by estimation of the logistic distribution parameters; see the covidSurv documentation for further computational details [26]. These analyses were applied separately to data on each age group to estimate separate fatality distributions, as well as to data on all age groups assuming the same time to death distribution over age, so that ${P}_{di}={P}_{d}$. Evidence of differences between age groups was then assessed by visual inspection and using the Cochrane Q-statistic for heterogeneity [29]. 

Using the basic relationship in Eq. (6), the fatality distribution can be estimated by either imposing the parametric model assumptions described above, or by leaving the model as a high-dimensional non-parametric model. In order to have a parsimonious and portable model that is easily summarised, the primary model was parametric. However, non-parametric analyses applied to separate age groups were also conducted to assess the validity of the parametric assumptions. Standard errors for the parameter estimates were obtained using 1000 bootstrap replications of the death counts, sampled with replacement from the observed data [23].

## Results

### Data summary

**Fig. 1:**
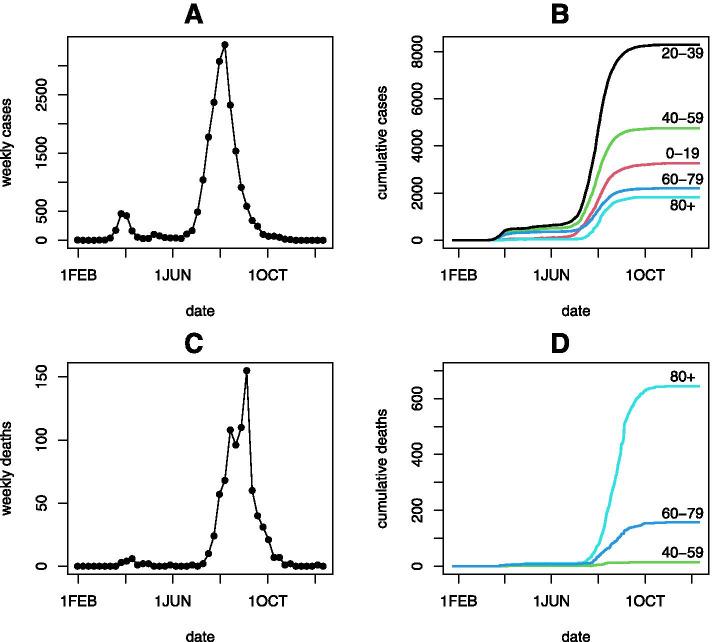
COVID-19 surveillance data for Victoria, Australia, 25 January—10 December 2020. Panels A and C depict weekly case and death counts, respectively. Panels B and D depict age-specific cumulative daily case and death counts, respectively

Victoria experienced two waves of COVID-19 cases during 2020, a smaller initial wave peaking in late March, and a larger second wave peaking in August. The pattern of new cases over time is depicted in Panel A of Fig. 1, with the pattern of new deaths displaying similar but lagged behaviour in Panel C of Fig. 1. At the end of the case series in Panel A there is a period of six consecutive weeks of zero cases ending in early December, signifying elimination of COVID-19 from the Victorian population at that point in time.

A summary of the age-specific counts of cases and deaths is displayed in Table 1, and is plotted in Panels B and D of Fig. 1. It is noteworthy that ages of 80 years and older had the least cases but yet the most deaths of all age groups. In total there were 820 deaths from 20,344 cases, however, deaths among cases younger than 50 years were very sparse, with only 4 deaths out of 14,123 cases observed. Since mortality was negligible for ages younger than 50 years, the primary analyses were applied to the 816 deaths among the 6235 cases aged 50 years or older. Age groups 50–59 years and 60–69 years had 14 and 28 deaths, respectively, and so analyses stratified into separate age groups were applied to the combined 42 deaths from 3504 cases in these two age groups combined (50–69 years).

**Table 1:** Age-specific COVID-19 cases and deaths in Victoria, Australia, during 25 January through 10 December, 2020. Observed case fatality risk (CFR) is the ratio of deaths to cases. Fitted CFR and 95% confidence intervals (CI) are from the fitted logistic CFR model

Age group (years)	Cases	Deaths	Observed CFR (%)	Fitted CFR (%)	95% CI (%)
0 – 9	1226	0	0	< 0.01	< 0.01
10 – 19	2035	0	0	< 0.01	< 0.01
20 – 29	4772	1	0.02	< 0.01	< 0.01
30 – 39	3525	2	0.06	0.01	0.003 – 0.03
40 – 49	2551	1	0.04	0.06	0.03 – 0.13
50 – 59	2205	14	0.63	0.46	0.26 – 0.69
60—69	1297	28	2.16	2.86	2.19 – 3.50
70 – 79	902	130	14.41	13.71	11.95 – 15.80
80 – 89	1094	349	31.90	32.00	29.71 – 34.17
> 90	737	295	40.03	40.04	36.63 – 43.57
Total	20,344	820	4.03	4.03	3.76 – 4.30

### Case fatality risk

The observed CFR by age group is displayed in Table 1. As explained in the Methods section, since COVID-19 infection was eliminated from the Victorian population by the end of the data series, the observed ratio of deaths to cases displayed in Table 1 is a valid estimate of CFR without the usual underestimation bias that occurs during an ongoing outbreak [21].

**Fig. 2:**
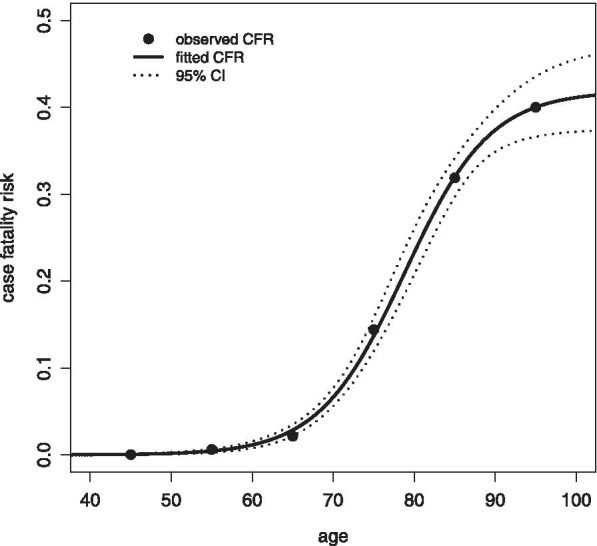
Observed and fitted age-specific COVID-19 case fatality risk (CFR) and 95% confidence interval (CI) for Victoria, Australia, 2020

The observed age-specific CFR values displayed a logistic pattern of age-dependence, as displayed in Fig. 2. The fitted CFR model specified by Eq. (2) is displayed in Fig. 2 where it is seen to be a good fit to the observed data. The fitted CFR values for each age group, together with the 95% confidence intervals are displayed in Table 1. Based on the model, CFR is estimated to have a steep age gradient, rising from 2.9% in the 60–69 years age group, to 40% in the age group age 90 years and older. Further discussion of the fitted CFR model and its contribution to the fatality distribution model is provided below.

### Time to death

**Fig. 3:**
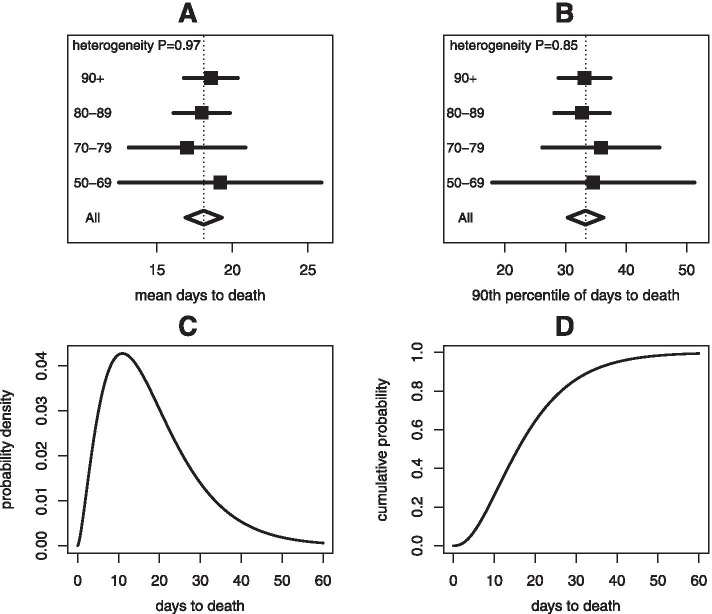
Estimated distribution of time between diagnosis and death among fatal COVID-19 cases. Panels A and B display the estimated means and 90% percentiles of the time to death, based on deconvolution analyses of the four age groups separately, and from the primary analysis of all cases over 50 years. Panels C and D display the probability density and the cumulative distribution functions of the time to death based on the primary analysis

A powerful feature of the analysis is the ability to estimate the distribution of the time from diagnosis to death, using data that have no link between diagnosis and death for individuals. Figure 3 displays the results of this analysis, based on the gamma model for the time to death.

Panels A and B of Fig. 3 provide the estimated mean and 90% percentile of the distribution, for each of the four age groups. The estimated mean time to death was similar across age, varying between 17 and 19 days for the four age groups with no evidence of heterogeneity (P = 0.97). Likewise, the estimated 90% percentile of the time to death distribution was similar across age, varying from 33 to 36 days for the four age groups with no evidence of heterogeneity (P = 0.85). Given the similar behaviour by age, the time to death distribution was fitted for all ages 50 years or older, assuming different CFR levels but the same time to death distribution. The estimated mean time to death was 18.1 days (95% CI: 16.9 – 19.3). The estimated 90% percentile of the distribution was 33.3 days (95% CI: 30.4 – 36.3). The fitted gamma model from this analysis is displayed in Panels C and D of Fig. 3.

### Age-specific fatality distribution

**Fig. 4:**
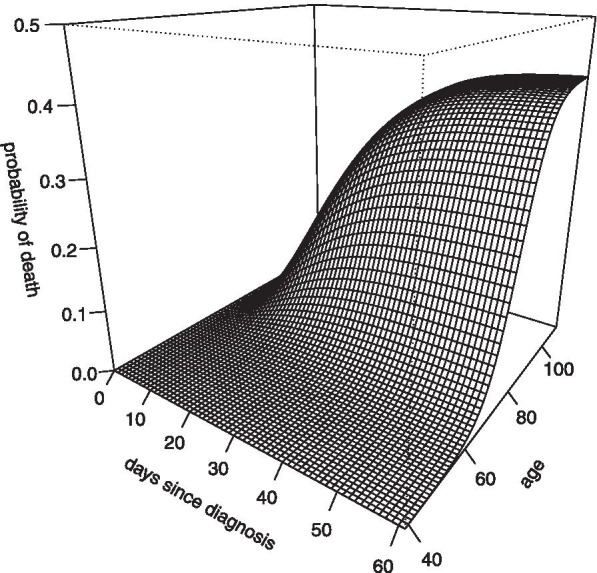
The model of the age-specific COVID-19 fatality distribution $F(t,a)$ as specified in Table 2. The plot depicts the cumulative probability of death within $t$ days of diagnosis for an individual aged $a$ years at diagnosis

Combining the two components of the fatality distribution in Eq. (4), leads to a three-dimensional function specifying the probability of death as a function of age and time since diagnosis. This distribution is plotted in Fig. 4 and the estimates of the parameters $({L}_{0},{L}_{1},{L}_{2},{G}_{1},{G}_{2})$ are displayed in Table 2. As well as providing key features of the natural history of COVID-19 infection, the full model specified in Table 2 may be used for age-specific adjustment of CFR in an ongoing outbreak, or non-age-specific adjustment using just the time to death distribution and an estimate of CFR averaged over the COVID-19 age distribution of the relevant population.

**Table 2:** Parameter estimates, standard errors and 95% confidence intervals (CI) for the fitted age-specific fatality distribution model, specified by Eqs. (2), (3) and (4). The final model has age dependent case fatality risk and age independent time to death distribution

	Case fatality risk			Time to death	
	${L}_{0}$	${L}_{1}$	${L}_{2}$	${G}_{1}$	${G}_{2}$
Estimate	0.4190	14.93	-0.1894	0.1403	2.540
Standard error	0.02500	1.057	0.01557	0.02168	0.3922
95% CI	(0.375,0.473)	(13.2,17.4)	(-0.225,-0.164)	(0.113,0.199)	(2.12,3.66)

### Model checking

**Fig. 5:**
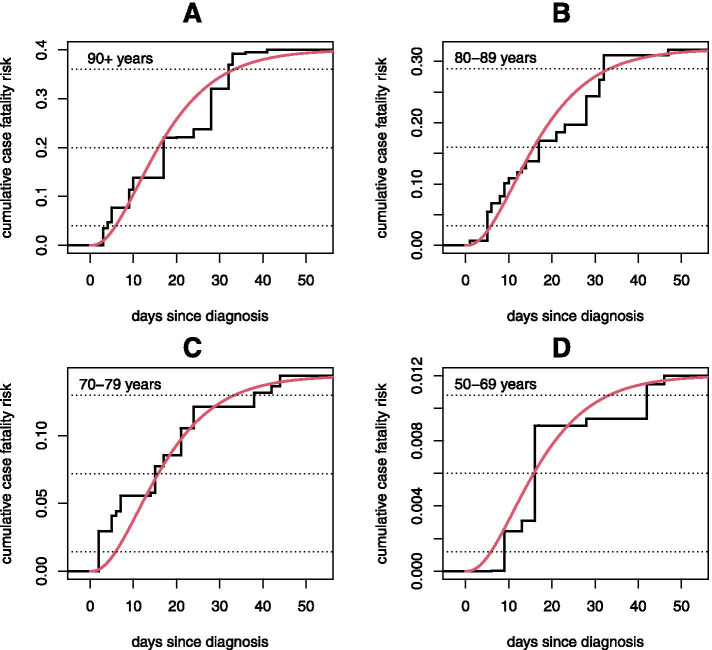
Comparison of the parametric age- and time-specific fatality model from Fig. 4 (red lines) with unrestricted non-parametric estimates obtained from each age group separately (black lines). Dotted lines denote 10%, 50% and 90% of the non-parametric CFR

The final model fitted to all four age groups was based on parametric assumptions, so non-parametric analyses within each separate age group were also conducted to check whether the parametric model provided a good fit. The non-parametric estimates are displayed in Fig. 5 for each of the four age groups. It is worth emphasising that these estimates, which are analogous to Kaplan–Meier estimates obtained from individual follow-up data, have been obtained without any linked diagnosis and death information at an individual level. Also displayed is the fitted parametric model applied to all data, as displayed in Fig. 4 and summarised in Table 2. The parametric model tracks the stratified non-parametric estimates excellently, demonstrating the validity of the parametric assumptions. In particular, it can be seen from the 50% percentile lines in Fig. 5 that the parametric model produces almost identical median survival times to the non-parametric models in each of the four age groups.

As a further model checking analysis, a simulation study was undertaken to explore whether the model fitting procedure was sensitive to departures from the assumed parametric assumptions. These simulations explored whether the assumed gamma model fit was adversely affected if the underlying distribution was actually a log-normal distribution. The results are presented in the Supplementary Information (Additional File 1) and provide evidence that the gamma model is robust to such departures from the assumptions.

## Discussion

Fatality risk is a key measure of disease burden and the fatality distribution quantifying time to death provides natural history information that is crucial for effective pandemic monitoring and control. There is extensive information about this distribution in widely available surveillance data and this paper has examined methods for extracting this information using statistical deconvolution. The basic idea is that the observed patterns of case diagnosis and death over age and time can be compared to reveal information about the quantity that links them, the fatality distribution.

By using surveillance data on case diagnosis and death, the study is an analysis of case fatality. This means that it is a study of mortality among individuals meeting the operational definition of a COVID-19 confirmed case. Infection fatality is an alternative concept to case fatality, referring to mortality among all infected individuals including those that do not meet the operational definition of a case [21]. Since surveillance data only capture information on confirmed cases, additional information, likely from prospective cohort studies, would be required to yield information about infection fatality and to assess whether it differs from case fatality. Nonetheless, case fatality is a key epidemiological measure of fundamental interest. Unlike the data set analysed in this paper, which is from a completed outbreak, most surveillance data sets globally contain active cases that may die in the future, so adjustment for right-censoring is usually a necessity when estimating CFR. The fatality distribution estimated in the current analysis is an appropriate estimate with which to adjust for such right-censoring when estimating CFR from surveillance data in ongoing outbreaks.

An implicit assumption of the analysis is that COVID-19 deaths reported in the surveillance data only occur among those who have been reported as a confirmed case. In other words, individuals whose death is captured by the surveillance system also have their positive status captured in the case series. This is likely to be a reasonable assumption for the Australian data but may need to be evaluated in other jurisdictions with reference to local information about how the surveillance system is organised. Note that this assumption does not preclude the possibility that some cases and deaths are unreported, which is likely to be a feature of most surveillance systems, but if a death is captured then a positive test would also need to have been captured. In general, the potential for infections to be unreported is a fundamental limitation of surveillance data that must be acknowledged. Nonetheless, surveillance data are a widely available and informative resource that allow immediate epidemiological information to be extracted while subsequent longer term prospective cohort studies are established and followed. In this sense they are a key resource that should be exploited during emerging outbreaks, while at the same time bearing in mind their potential limitations.

The age-specific data from Victoria were available from an outbreak that culminated in elimination of the virus, providing a valuable opportunity to develop a model of age-specific mortality. Evaluation of mortality in an age-specific framework is highly desirable because the age distribution of an outbreak is likely to be specific to the population and it may change over time. This would mean that an age-aggregated analysis of CFR could be skewed by the particular age distribution of the population. In the Victorian data there was strong evidence that the age distribution evolved over time, with the first wave having 2.7% of cases older than 80 years compared to 9.5% in the second wave. This lead to an increase over time in the age-aggregated CFR, and underlines the need for age-specific analysis.

## Conclusions

Deconvolution provides the ability to estimate the distribution of the delay between COVID-19 diagnosis and death, using data that have no linkage between diagnosis and death at an individual level. It is therefore a powerful analysis tool that could be widely applied to extract rich mortality information from existing global surveillance data.

The primary output of the analysis presented here is an age-specific mortality model specified by Eqs. (2), (3) and (4), summarised in Table 2 and plotted in Fig. 4. The model estimates a mean time from diagnosis to death of 18 days, with a standard deviation of 11 days and 90% percentile of 33 days. Previous studies based on clinical cohorts have reported mean time to death ranging from 10 to 20 days [2–5]. Modelling used by the Australian government employs the epiforecasts [30] platform for calibration, which assumes a time to death distribution with mean 13 days, based on data from Wuhan, China [8, 9]. The analysis of Australian data presented here suggests that a longer delay to death may be more appropriate for modelling in the Australian context.

The fitted fatality distribution model can be used in various ways. Assumptions about time delays and fatality risk are essential for developing mathematical models of transmission dynamics and so the fitted model can inform this process [16, 31, 32]. Furthermore, delay-adjusted estimates of CFR during an emerging outbreak often use an external estimate of the time to death distribution to account for the fact that recent cases are not yet resolved [13–15]. Although it has been argued that external estimates of time to death are unnecessary for this purpose [23], they are nonetheless commonly employed and the model presented here could be used. Another use is for short-term projection of future mortality using the convolution relationship in Eq. (4) together with the observed cases series, as has been used for short-term projection of future cases using an estimate of the incubation period distribution [7]. All of these uses highlight the value of a parsimonious specification of mortality dynamics for COVID-19. Future research should consider the application of this modelling strategy to global surveillance data.

## Supplementary Information

**Additional file 1. **Simulations of the effect of departures from the parametric assumptions.

## Data Availability

The datasets and software used in the current study are available in a GitHub repository at https://www.github.com/ianmar/covidSurv as part of the R package covidSurv version 0.1.0. The package includes data frames covidCasesAge and covidDeathsAge that contain the daily case and death counts by age group. As explained in the paper, these datasets were constructed from publically available age-specific line listings of cases and deaths available at https://www.dhhs.vic.gov.au/victorian-coronavirus-covid-19-data and https://www.covid19data.com.au/deaths, respectively.
