# Supplementary material for: Estimating age-specific COVID-19 fatality risk and time to death by comparing population diagnosis and death patterns: Australian data
Source: BMC Med Res Methodol. 2021 Jun 21;21:126. doi: 10.1186/s12874-021-01314-w (PMC8215490; doi:10.1186/s12874-021-01314-w)
Supplement: Supplementary file 1 — Additional file 1. Simulations of the effect of departures from the parametric assumptions. [file 12874_2021_1314_MOESM1_ESM.pdf]

**Supplementary Information for the paper "Estimating age-specific COVID-19 fatality risk and time to death by comparing population diagnosis and death patterns: Australian data"**

**Ian C. Marschner**
**NHMRC Clinical Trials Centre**
**University of Sydney**

In this Supplementary Information we consider a simulation study providing a sensitivity analysis for the assumed gamma distribution parametric model of the time between diagnosis and death. The gamma model has been the most commonly employed model for the time to death among COVID-19 infected individuals [5,6,15]. However, other models have also been employed, most notably the log-normal distribution [6].

The simulation study presented here considered how the gamma model performs when the time to death distribution is actually a log-normal distribution. This is intended to explore the robustness of the model fitting method.

## Calibration of the log-normal model

The simulations used a log-normal time to death distribution with mean and variance that was calibrated to the fitted gamma model presented in Panels C and D of Figure 3 of the main paper. This allows assessment of whether departures from the shape of the gamma distribution adversely affect the performance of the model fitting method. Figure S1 below compares the shape of the assumed log-normal distribution to the gamma model with the corresponding parameter values of the two distributions displayed.

**Figure S1** Gamma and log-normal models

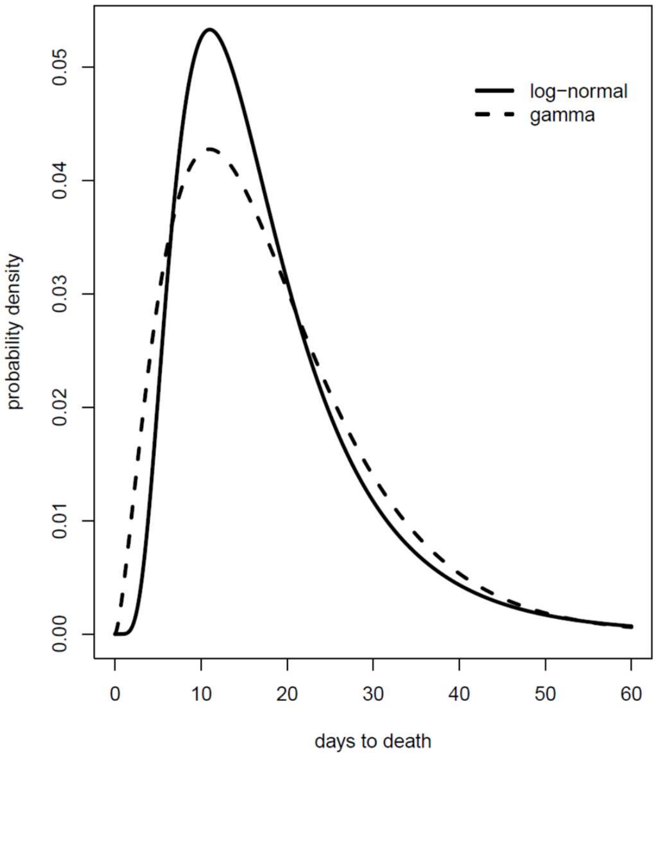


Figure S1 displays the probability density of the log-normal distribution

$$f(t) = \frac{1}{t\sigma\sqrt{2\pi}} \exp\left(-\frac{(\log t - \mu)^2}{2\sigma^2}\right)$$

with parameters $\mu = 2.730$ and $\sigma = 0.5762$ and compares it with the probability density of the gamma distribution

$$f(t) = \frac{{G_1}^{G_2}}{\Gamma(G_2)} t^{G_2 - 1} \exp(-G_1 t)$$

with parameters $G_1 = 0.1403$ and $G_2 = 0.2540$

as in the fitted gamma model reported in Table 2 of the main paper. Both distributions have mean 18.10 days and variance 129.04 days.

## Simulations

Simulated data were generated by adding log-normal simulated survival times onto the diagnosis times of the observed case series, in each age group, with the risk of death being the same as the observed CFR within that age group. This was conducted 100 times, which produced 100 simulations of the daily death count series. The 100 simulations of the death count series were analysed together with the observed case count series, by applying the gamma model fitting method to each simulation. The characteristics of the fitted gamma distribution were then compared with the characteristics of the true underlying log-normal distribution.

## Results

The results of the simulations are presented in Table S1. Based on the 100 fitted gamma distributions, the average of the 100 estimates of the mean time to death was 18.86 days, compared to a true value of 18.10 days. The average of the 100 estimates of the variance of the time to death was 130.42 days, compared to a true value of 129.04 days. Thus, the location and spread of the fitted gamma distribution was very similar to that of the true underlying log-normal distribution. Other characteristics of the fitted gamma distributions were also similar to the true underlying log-normal distribution, including the 90% percentile of the distribution, which had an average of 33.46 days in the 100 simulations, compared to a true value of 32.09 days.

**Table S1 Simulation results for estimating the time to death distribution**

| | True value | Simulated average | Simulated bias |
|---|---|---|---|
| mean | 18.10 | 18.86 | 4.2% |
| variance | 129.04 | 130.42 | 1.1% |
| 90% percentile | 32.09 | 33.46 | 4.1% |

## Conclusions

The simulations results provide evidence that when the distribution has a non-gamma form, specifically a log-normal form, the gamma model fit still recovers key features of the distribution in an unbiased way. This is indicative of the model fitting method being robust to departures from the assumed parametric form, at least to the extent afforded by the log-normal distribution. These results are consistent with the discussion in the main paper, where it was argued that the unlinked surveillance data considered in this paper are unlikely to have the sensitivity to differentiate between unimodal right-skewed positive distributions for the time to death, including the gamma, log-normal and other similar distributions.
